# Efficacy of matrilin-3-primed adipose-derived mesenchymal stem cell spheroids in a rabbit model of disc degeneration

**DOI:** 10.1186/s13287-020-01862-w

**Published:** 2020-08-24

**Authors:** Manjunatha S. Muttigi, Byoung Ju Kim, Hemant Kumar, Sunghyun Park, Un Yong Choi, Inbo Han, Hansoo Park, Soo-Hong Lee

**Affiliations:** 1 School of Integrative Engineering, Chung-Ang University, Seoul, 06911 South Korea; 2 Department of Medical Biotechnology, Dongguk University-Seoul, Seoul, 04620 South Korea; 3 Department of Pharmacology and Toxicology, National Institute of Pharmaceutical Education and Research (NIPER)-Ahmedabad, Gandhinagar, Gujarat 382010 India; 4 Department of Biomedical Science, CHA University, Seongnam-si, 13488 South Korea; 5 Department of Neurosurgery, School of Medicine, CHA Bundang Medical Center, CHA University, Seongnam-si, 13496 South Korea

**Keywords:** Intervertebral disc, Degeneration, Mesenchymal stem cell, Matrilin-3, Priming, Spheroid, Engineered MSCs

## Abstract

**Background:**

Chronic low back pain is a prevalent disability, often caused by intervertebral disc (IVD) degeneration. Mesenchymal stem cell (MSC) therapy could be a safe and feasible option for repairing the degenerated disc. However, for successful translation to the clinic, various challenges need to be overcome including unwanted adverse effects due to acidic pH, hypoxia, and limited nutrition. Matrilin-3 is an essential extracellular matrix (ECM) component during cartilage development and ossification and exerts chondrocyte protective effects.

**Methods:**

This study evaluated the effects of matrilin-3-primed adipose-derived MSCs (Ad-MSCs) on the repair of the degenerated disc in vitro and in vivo. We determined the optimal priming concentration and duration and developed an optimal protocol for Ad-MSC spheroid generation.

**Results:**

Priming with 10 ng/ml matrilin-3 for 5 days resulted in the highest mRNA expression of type 2 collagen and aggrecan in vitro. Furthermore, Ad-MSC spheroids with a density of 250 cells/microwell showed the increased secretion of favorable growth factors such as transforming growth factor beta (TGF-β1), TGF-β2, interleukin-10 (IL-10), granulocyte colony-stimulating factor (G-CSF), and matrix metalloproteinase 1 (MMP1) and decreased secretion of hypertrophic ECM components. In addition, matrilin-3-primed Ad-MSC spheroid implantation was associated with optimal repair in a rabbit model.

**Conclusion:**

Our results suggest that priming MSCs with matrilin-3 and spheroid formation could be an effective strategy to overcome the challenges associated with the use of MSCs for the treatment of IVD degeneration.

## Background

Intervertebral disc (IVD) degeneration is one of the main causes of chronic low back pain (LBP), leading to disability and increasing financial burden [1–3]. Discogenic LBP is the most common type of chronic LBP, representing 26–42% of affected cases [1, 2]. The degeneration of the disc causes a shift from type II to type I collagen expression by nucleus pulposus (NP) cells, and a decrease in aggrecan synthesis leads to dehydrated discs with the loss of swelling pressure required for mechanical support [1, 4, 5].

Although a variety of surgical and non-surgical procedures have been developed to treat discogenic LBP, there has been increased interest in regenerative medicine due to the limitation in current treatments. Based on reported experimental and clinical studies, mesenchymal stem cell (MSC) therapy has been considered to be a safe and feasible option for repairing the degenerated disc in patients in the early degeneration stage [1, 4, 6–20]. However, for successful translation to the clinic, various challenges need to be overcome due to the harsh disc environment such as low cellularity, low glucose, low oxygen, acidic pH, limited nutrients under an inflammatory milieu, and possible osteophyte formation by transplanted cells [1, 2, 21]. There have been several studies to develop better strategies to promote disc repair by MSCs, including hypoxia pretreatment of MSCs, co-culture of MSCs with NP cells, and combined use of scaffolds [9–13, 16, 17].

In this study, we focused on matrilin-3, an extracellular matrix (ECM) component essential for cartilage development and ossification, because it can exert chondroprotective effects [22–26]. Matrilin-3 acts as an adapter protein and plays a major role in the formation of filamentous networks [22, 23]. Mutations in matrilin-3 (R116W and C299S) have been found to lead to an aberrant response towards TGF-β signaling and the early hypertrophic differentiation of chondrocytes [24]. Matrilin-3 protein binds with BMP-2, thereby preventing downstream signaling and the hypertrophy of chondrocytes [26]. In addition, clinical evidence suggests that mutations in matrilin-3 could lead to spondyloepimetaphyseal dysplasia, multiple epiphyseal dysplasia, early onset of osteoarthritis, and IVD degeneration [27–29]. Furthermore, recombinant matrilin-3 protein could enhance the gene expression of type 2 collagen and aggrecan in chondrocytes, synovial fibroblasts, and Ad-MSCs [24, 30]. In our previous study, the co-administration of recombinant matrilin-3 with Ad-MSCs led to hyaline-like cartilage regeneration in a rat osteochondral defect model [30].

In the present study, we hypothesized that matrilin-3 priming to Ad-MSCs could enhance the secretion of growth factors and cytokines, which are favorable for the repair of the degenerated disc and suppression of hypertrophic differentiation and calcification. To investigate our hypothesis, we first optimized the matrilin-3 priming concentration and duration and determined an optimal protocol for Ad-MSC spheroid generation. Second, we evaluated the therapeutic potency of matrilin-3-primed Ad-MSC spheroids in the regeneration of degenerated NP (dNP) cells in vitro. Finally, we investigated whether matrilin-3-primed Ad-MSCs could improve Ad-MSC function and enhance the regenerative effect in a rabbit model of disc degeneration.

## Methods

### Isolation and culture of Ad-MSCs from adipose tissue

Infrapatellar fat pads were harvested during total knee arthroplasty and used for Ad-MSC isolation after obtaining informed consent and institutional ethics committee (IRB number: IACUC150058) approval [30]. In brief, the infrapatellar fat pads were washed with phosphate-buffered solution (PBS), rinsed, and chopped into small pieces. The debris was removed, and type II collagenase (1 mg/ml) was added. Digestion was aided by pipetting up and down with a 25 ml pipette. The digested samples were collected in 50 ml falcon tubes and incubated in a warm water bath (37 °C) for 15 min. After incubation, the samples were minced again and incubated in a shaking incubator for a further 15 min. The samples were finally centrifuged at 1000 rpm for 10 min, and the surfactant layer was removed. The retained pellet was resuspended in the following medium: Dulbecco’s modified Eagle medium–Low Glucose (DMEM-LG; HyClone, GE Healthcare Life Sciences, Logan, UT, USA) + 10% fetal bovine serum (FBS; HyClone, Australian origin, GE Healthcare Life Sciences, Logan, UT, USA) + 0.1 mg/ ml streptomycin + 100 units/ml penicillin. The resuspended samples were passed through a sterile filter (40 μm) and centrifuged again at 1000 rpm for 10 min. The pellet containing the stromal vascular fraction was suspended in culture medium and incubated in humidified air with 5% CO_2_ at 37 °C. The medium was changed every 2 days. If cells covered more than 70% of the culture flask, the Ad-MSCs were detached with 0.05% trypsin and re-cultured at a seeding density of 6.5 × 10^3^ cells/cm^2^. Passage 4 cells were used for in vitro and in vivo experiments.

### Isolation of human NP cells

Human lumbar IVD tissues [to-be-discarded surgical waste, approved after review by our institute’s ethics committee (IRB No. 2014-07-097)] were obtained from patients undergoing surgery for degenerative disc disease. Specifically, NP cells were isolated from patients undergoing lumbar discectomy (L3/4) for lumbar disc herniation (L3/4) and lumbar stenosis (L3/4). Patients were graded as a Pfirrmann grade III according to T2 MRI results. Disc tissues were rinsed with Dulbecco’s phosphate-buffered saline (DPBS; HyClone, GE Healthcare Life Sciences, Logan, UT, USA) containing 1% penicillin and streptomycin (Gibco BRL, Grand Island, NY, USA) three times for 15 min and grossly separated into the annulus fibrosus and NP. The separated NP tissue samples were digested with 0.05% (w/v) type 2 collagenase (Sigma-Aldrich, St. Louis, MO, USA) for 6 h. The digested mixture was strained with a cell strainer (40 μm pore size; Becton Dickinson, Franklin Lakes, NJ, USA), centrifuged for 5 min at 1000 rpm, and washed with DPBS twice to remove the remaining collagenase. The cells were suspended in DMEM-LG supplemented with 10% FBS, 0.1 mg/ml streptomycin, and 100 units/ml penicillin and cultured until they were 85% confluent. Passage 2 cells were used for co-culture experiments.

### Priming of Ad-MSCs with recombinant matrilin-3: duration and concentration optimization

Ad-MSCs were seeded at a density of 1500 cells/cm^2^ on cell culture plates and incubated at 37 °C with 5% CO_2_. After 12 h of seeding, the culture medium was changed to serum starvation medium (DMEM-LG and 1× penicillin and streptomycin) and incubated for 12 h in a CO_2_ incubator. After 12 h of serum starvation, the culture medium was supplemented with 10 ng/ml (group 2), 20 ng/ml (group 3), or 50 ng/ml (group 4) of matrilin-3. Ad-MSCs cultured in the medium without matrilin-3 supplementation served as the control (group 1). The culture medium was changed every 24 h with fresh matrilin-3 for 5 days. Ad-MSCs on days 1, 3, and 5 were evaluated by live/dead assay, Cell Counting Kit-8 (CCK-8) staining, and mRNA expression analysis of sex-determining region Y (SRY)-box 9 (SOX9), type 2 collagen, and aggrecan.

### Ad-MSC culture condition optimization: monolayer and spheroid preparation

To optimize culture conditions, the following six groups were used: group 1, Ad-MSC monolayer; group 2, matrilin-3-primed Ad-MSC monolayer; group 3, Ad-MSC spheroids (seeding density: 3 × 10^5^ cells/2400 microwells); group 4, matrilin-3-primed Ad-MSC spheroids (seeding density: 3 × 10^5^ cells/2400 microwells); group 5, matrilin-3-primed Ad-MSC spheroids (seeding density: 6 × 10^5^ cells/2400 microwells); and group 6, matrilin-3-primed Ad-MSC spheroids (seeding density: 12 × 10^5^ cells/2400 microwells). The spheroids were generated using EZSPHERE Microplate 6 well (well size: 400–500 μm diameter; 100–200 μm depth; 2400 microwells/9.6 cm^2^) (Reprocell Inc., Yokohama, Japan). In brief, 6-well plates were washed with DPBS to removes air bubbles. Then, the cells were seeded at a seeding density of 3 × 10^5^ (groups 3 and 4), 6 × 10^5^ (group 5), or 12 × 10^5^ (group 6) in 3 ml of culture medium (DMEM-LG, 10% FBS, 1× penicillin and streptomycin) for each well. After seeding, cell suspensions were gently mixed with a 1 ml pipette to remove air bubbles and to ensure uniformity. Culture plates were incubated for 24 h in a CO_2_ incubator. After 24 h, spheroids were collected in wide orifice 1 ml pipette tips. The spheroids were washed with DPBS, gently centrifuged using a microcentrifuge, and used for live/dead staining, p53 and BAX mRNA expression analysis, and cytokine array analysis.

### Co-culture method

Indirect co-culture methods were used with a Transwell culture system (Becton Dickinson, Franklin Lakes, NJ, USA). The effects of primed Ad-MSC secretome on the regeneration of dNP cells were evaluated with the following groups: group 1, dNP cells only; group 2, dNP cells + Ad-MSC spheroids; group 3, dNP cells + matrilin-3-primed Ad-MSC monolayer; and group 4, dNP cells + matrilin-3-primed Ad-MSC spheroids. In brief, dNP cells were cultured in 6-well plates at a seeding density of 75,000 cells/well, and the Ad-MSC spheroid or monolayer was seeded at a density of 3 × 10^5^ on inserts (3 μm pore membrane) (Corning Inc., Corning, NY, USA). The indirect cultures were maintained in a culture medium containing DMEM-LG + 10% FBS + 1× penicillin and streptomycin. Every 2 days, the culture medium was changed, and RNA samples were collected on day 5 and day 10. The immunofluorescence was analyzed on day 10 for the expression of chondrogenic, hypertrophic, and ossification markers.

### Cytotoxicity and proliferation assays

A live/dead assay (Life Technologies, Seoul, Korea) was performed as per the supplier’s instructions, and images of the cells were captured on days 1, 3, and 5 using the Cytation 3 Cell Imaging Multi-Mode Reader (Biotek Instruments, Inc., Winooski, VT, USA). Cell proliferation was assessed on days 1, 3, and 5 by CCK-8 staining (Dojindo Molecular Technologies, Inc., Rockville, MD, USA) as per the supplier’s instructions.

### RNA extraction and mRNA expression analysis

Real-time quantitative polymerase chain reaction (RT-PCR) was performed according to standard procedures. In brief, total RNA was extracted using a TRIzol kit (Thermo Fisher Scientific, Inc., Waltham, MA, USA). Complementary DNA was subsequently prepared using 0.5 μg of RNA with the PrimeScript™ RT reagent kit (Takara Bio Inc., Shiga, Japan), and RT-PCR amplification was performed using the StepOnePlus Real-Time PCR System. For each target gene, relative mRNA expression levels were calculated using the 2^−ΔCt^ method with the expression level of 18S RNA as an internal control. The target primers are listed in Table 1.

**Table 1:** Primers used for RT-PCR

Gene	Primer sequence	Accession number	Amplicon (bp)
18S	5′-GTA ACC CGT TGA ACC CCA TT-3′ 5′-CCA TCC AAT CGG TAG TAG CG-3′	NR_003286.2	151
SOX9	5′-GTA CCC GCA CTT GCA CAA C-3′ 5′- TCT CGC TCT CGT TCA GAA GTC-3′	NM_000346.3	74
Collagen 2A	5′- GGGAGTAATGCAAGGACCA-3′ 5′- ATCATCACCAGGCTTTCCAG-3′	NM_001844.4	175
Aggrecan	5′-GCC TGC GCT CCA ATG ACT-3′ 5′-ATG GAA CAC GAT GCC TTT CAC-3′	NM_013227.3	104
Collagen A1	5′-CCC CTG GAA AGA ATG GAG ATG-3′ 5′-TCC AAA CCA CTG AAA CCT CTG-3′	NM_000088.3	148
MMP13	5′-TCA CCA ATT CCT GGG AAG TCT-3′ 5′-TCA GGA AAC CAG GTC TGG AG-3′	NM_002427.3	95
Collagen 10	5′-ACG CTG AAC GAT ACC AAA TG-3′ 5′-TGC TAT ACC TTT ACT CTT TAT GGT GTA-3′	NM_000493.3	101
p53	5′-GGCCCACTTCACCGTACTAA-3′ 5′-GTGGTTTCAAGGCCAGATGT-3′	NM_000546	156
BAX	5′-TTTGCTTCAGGGTTTCATCC-3′ 5′-CAGTTGAAGTTGCCGTCAGA-3′	NM_001291428.1	246

### Cytokine array

The expression levels of cytokines, growth factors, and matrix proteases in non-primed and primed Ad-MSCs with matrilin-3 were detected using Custom Sandwich-based Antibody Array (RayBiotech Inc., Norcross, GA, USA). The list of growth factors and cytokines are shown in Table 2. In brief, the Ad-MSC monolayer (3 × 10^5^ cells) was incubated in 2 ml of culture medium containing DMEM-LG and 1× penicillin and streptomycin for 2 days. Similarly, spheroids (3 × 10^5^ cells) were seeded in ultra-low attachment plates with 2 ml of culture medium containing DMEM-LG and 1× penicillin and streptomycin for 2 days. After 2 days, the conditioned medium was collected, and cytokine array analysis was performed as per the manufacturer’s instructions. Immunoreactivity was detected using the ChemiDoc™ XRS+ detection system (BIORAD iNtRON Biotechnology, Seoul, Korea). The signal densities for each protein were semi-quantitatively analyzed using Image Lab software (Bio-Rad, Hercules, CA, USA) and normalized to the positive control of each sample.

**Table 2:** RayBiotech’s Custom Sandwich-based Antibody Array design

	A	B	C	D	E	F	G	H	I	J	K	L
**1**	**POS**	**POS**	**NEG**	**NEG**	**TGF-β1**	**TGF-β2**	**TGF-β3**	**SDF-1**	**bFGF**	**EGF**	**G-CSF**	**CCL5**
**1**	**POS**	**POS**	**NEG**	**NEG**	**TGF-β1**	**TGF-β2**	**TGF-β3**	**SDF-1**	**bFGF**	**EGF**	**G-CSF**	**CCL5**
**2**	**IL-11**	**IL-1β**	**IL-6**	**MMP1**	**MMP9**	**TNF-α**	**TIMP-1**	**TIMP-2**	**HGF**	**VEGF**	**IGF-1**	**GDF-15**
**2**	**IL-11**	**IL-1β**	**IL-6**	**MMP1**	**MMP9**	**TNF-α**	**TIMP-1**	**TIMP-2**	**HGF**	**VEGF**	**IGF-1**	**GDF-15**
**3**	**BMP-2**	**BMP-7**	**BMP-9**	**Adipsin**	**MMP13**	**Activin-A**	**IL-4**	**IL-10**	**MATN3**	**IL-1ra**	**BLANK**	**POS**
**3**	**BMP-2**	**BMP-7**	**BMP-9**	**Adipsin**	**MMP13**	**Activin-A**	**IL-4**	**IL-10**	**MATN3**	**IL-1ra**	**BLANK**	**POS**

### Immunocytochemistry

For immunofluorescence microscopy, dNP cells co-cultured with primed and non-primed Ad-MSCs for 10 days were used. These cells were fixed for 10 min with 4% paraformaldehyde (PFA) in PBS at room temperature, washed three times with 1× PBS, and permeabilized with 0.5% Triton-X for 10 min. The cells were washed with PBS and blocked for 45 min in blocking buffer (5% BSA and 0.5% Tween-20 in 1× PBS) containing 10% normal goat serum (Gibco, New Zealand origin) at room temperature. For immunostaining, the cells were incubated overnight at 4 °C with cadherin-2 (1:200, Abcam), chondroitin sulfate (1:100, Abcam), and collagen 1 (1:200, Abcam). Then, they were incubated with the secondary antibodies goat anti-rabbit Alexa Fluor® 568 and goat anti-mouse Alexa Fluor® 488 (Abcam) for 1 h at room temperature. The cells were counterstained with DAPI (Vector Laboratories, Burlingame, CA, USA), and images were acquired using Cytation 3 Cell Imaging Multi-Mode Reader (Biotek Instruments, Inc., Winooski, VT, USA). The detected fluorescence intensities were used to analyze the expression.

### Annulus needle puncture model of IVD degeneration in rabbits and matrilin-3-primed Ad-MSC spheroid implantation

All animal procedures were performed in accordance with a protocol approved by the Institutional Animal Care and Use Committee (IACUC) of our institute (IACUC160058). New Zealand White rabbits (4~5 months old, approximately 3~3.5 kg in weight) were raised at 55–65% humidity and a controlled temperature of 24 ± 3 °C with a light/dark cycle of 12 h. The rabbits had free access to food and tap water ad libitum. The rabbits were anesthetized by intramuscular injection of tiletamine hydrochloride/zolazepam hydrochloride (Zoletil, 50 mg/kg; Virbac Laboratories, Carros, France) and xylazine (Rompun, 10 mg/kg; Bayer, Seoul, Korea). IVD degeneration was induced by annular needle puncture via a retroperitoneal approach, as previously reported [15, 31]. In brief, the right flank was shaved from the ventral to dorsal midline and from the 12th rib to the iliac crest. The rabbit was then positioned in the right lateral decubitus position. The skin was prepared for aseptic surgery with povidone iodine and alcohol. A 6-cm oblique skin incision was made between the right 12th rib and right iliac crest, approximately halfway between the dorsal and ventral midline. After the incision of skin and subcutaneous tissues, the external oblique muscle was incised approximately 2 cm ventral to the junction of the fascia covering the paraspinal musculature and the longitudinally running fibers of the external oblique. The IVDs of L3/4 and L4/5 were then exposed between the psoas and abdominal cavity ventral and the paraspinal muscles dorsally by careful paraspinal muscle dissections. The IVDs of L3/4 and L4/5 were punctured to a depth of 5 mm using an 18G spinal needle, and the needle was rotated 360°.

After 1 week of needle puncture, a volume of 100 μl (2 × 10^6^ cells/100 μl PBS) was slowly injected into the center of the IVD using a 22G spinal needle via a left lateral retroperitoneal approach. The rabbits (*n* = 35) were divided into five groups (*n* = 7/group) to determine the effects of matrilin-3-primed Ad-MSC spheroids on optimal disc repair (group A: L3/4 and L4/5 discs were degenerated with the L5/6 disc as the normal control; group B: L3/4 and L4/5 discs were degenerated with Ad-MSC implantation; group C: L3/4 and L4/5 discs were degenerated with matrilin-3-primed Ad-MSC implantation; group D: L3/4 and L4/5 discs were degenerated with Ad-MSC spheroid implantation; group E: L3/4 and L4/5 discs were degenerated with matrilin-3-primed Ad-MSC spheroid implantation). After 13 weeks of the initial annular puncture (12 weeks after implantation), all rabbits were sacrificed to analyze the effects of matrilin-3-primed Ad-MSC spheroids on the repair of the degenerated disc.

### MRI analysis of IVDs

T2 MRI was performed to confirm the induction of IVD degeneration. A 4.7T MRI spectrometer (Bruker Biospec 47/40) and a custom MR coil were used for coronal and sagittal T2 MRI with the following settings: time to repetition of 3200 ms, time to echo of 130 ms, 320 (horizontal) × 320 (vertical) matrix, field of view of 120°, and 2 mm slices with 0.2 mm spacing between each slice. T2 MRI of all groups was performed to evaluate disc water content changes at 12 weeks after transplantation. The mean T2 signal was calculated for the annulus fibrosus and the NP of all discs. The T2 signal intensity of whole discs was measured using ImageJ software.

### Histological analysis

All rabbits were euthanized with CO_2_ inhalation, and the L1–L6 vertebral bodies from all rabbits were fixed in 4% PFA. The samples were decalcified using RapidCal Immuno (BBC Biochemical, Mount Vernon, WA, USA). The tissues were dehydrated in a graded series of ethanol and xylene and embedded in paraffin, and axial sections (5 μm thickness) were obtained. The sections were stained with safranin-O/fast-green for proteoglycan accumulation within the newly formed matrix.

### Statistical analysis

The results are reported as the mean ± standard error of the mean (SEM), and differences with *p* values < 0.05 were considered statistically significant. Two-way analysis of variance (ANOVA) followed by Tukey’s *post hoc* test was used to compare the mean values among groups.

## Results

### Matrilin-3 priming dose and duration selection

**Fig. 1:**
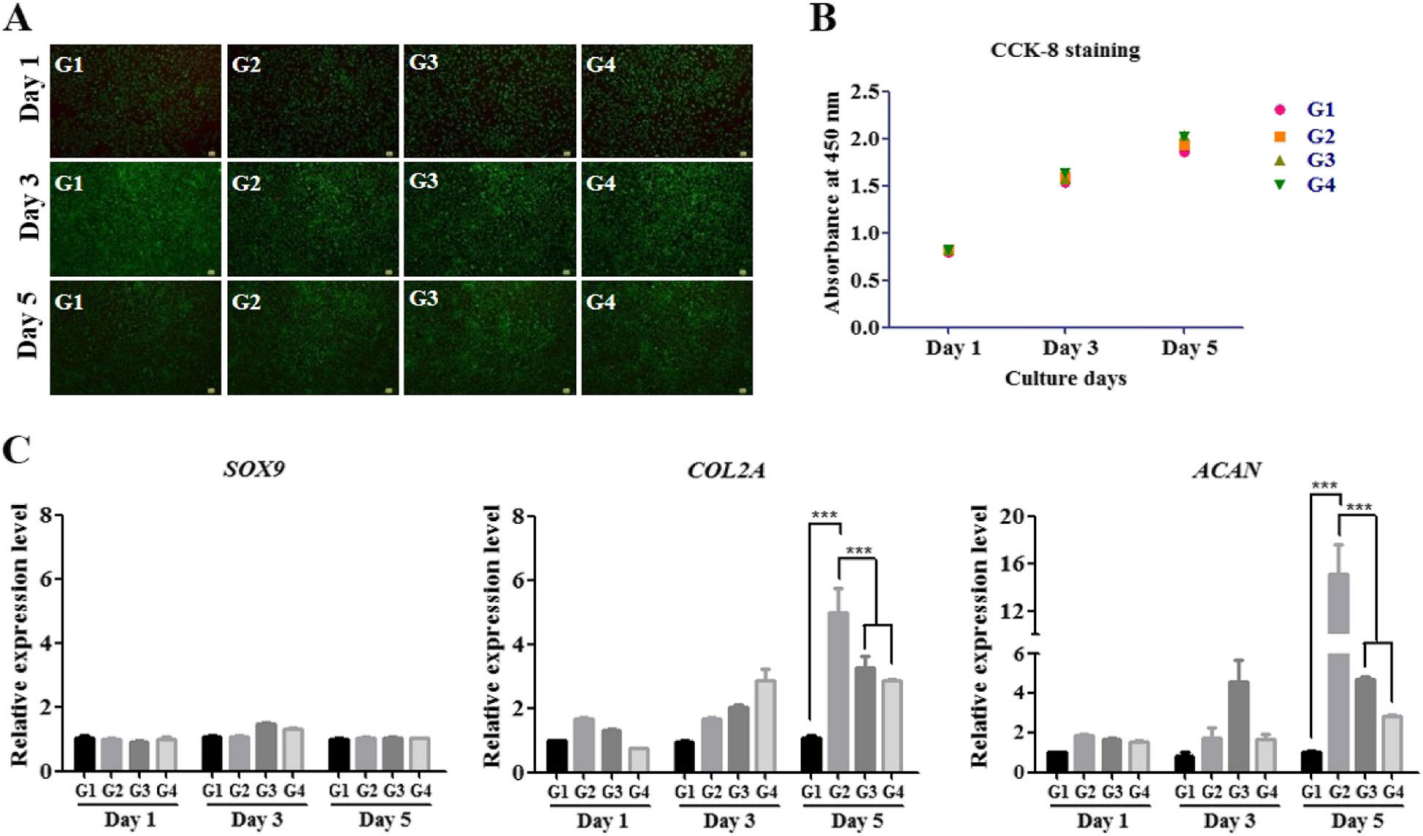
Matrilin-3 priming dose and duration selection. **a** Live/dead assay. **b** Proliferation assay. **c** mRNA expression of the chondrogenic markers SOX9, collagen 2, and aggrecan. G1, Ad-MSCs; G2, Ad-MSCs + matrilin-3 (10 ng/ml); G3, Ad-MSCs + matrilin-3 (20 ng/ml); G4, Ad-MSCs + matrilin-3 (50 ng/ml). Statistically significant expression at *p < 0.05, **p < 0.01, ***p < 0.001

Ad-MSCs were treated with different concentrations of matrilin-3 (10, 20, or 50 ng/ml) and cultured for different days (1 day, 3 days, or 5 days). Priming with matrilin-3 did not alter Ad-MSC morphology compared with the morphology of non-primed cells. Live/dead assay (Fig. 1a) and CCK-8 staining assay (Fig. 1b) indicated no cytotoxicity, and the proliferation rate of Ad-MSCs was not affected after priming with matrilin-3. Flow cytometric analysis of matrilin-3-primed Ad-MSCs identified MSC surface markers such as CD105, CD90, and CD73 (Supplementary Fig. 1). These results demonstrated that matrilin-3 priming to Ad-MSCs did not affect viability and proliferation. The mRNA expression level of SOX9, a master transcription factor for chondrogenesis, was not significantly different during priming with matrilin-3 (Fig. 1c). The mRNA expression levels of collagen 2 and aggrecan were significantly increased in matrilin-3-primed Ad-MSCs at 10, 20, and 50 ng/ml (Fig. 1c). Collagen 2 mRNA expression levels showed a dose-dependent upregulation on culture day 3. On the other hand, both collagen 2 and aggrecan expression levels were higher in matrilin-3-primed Ad-MSCs at 10 ng/ml. However, a dose-dependent decrease in the mRNA expression of collagen 2 and aggrecan was observed at 20 ng/ml and 50 ng/ml on culture day 5. These results demonstrated that matrilin-3 regulation of collagen 2 and aggrecan expression may depend on the dose and duration of priming. Based on the mRNA expression of collagen 2 and aggrecan, we selected a culture duration of 5 days and 10 ng/ml matrilin-3 for further experiments (***p < 0.001).

### Matrilin-3-primed Ad-MSC culture condition optimization

**Fig. 2:**
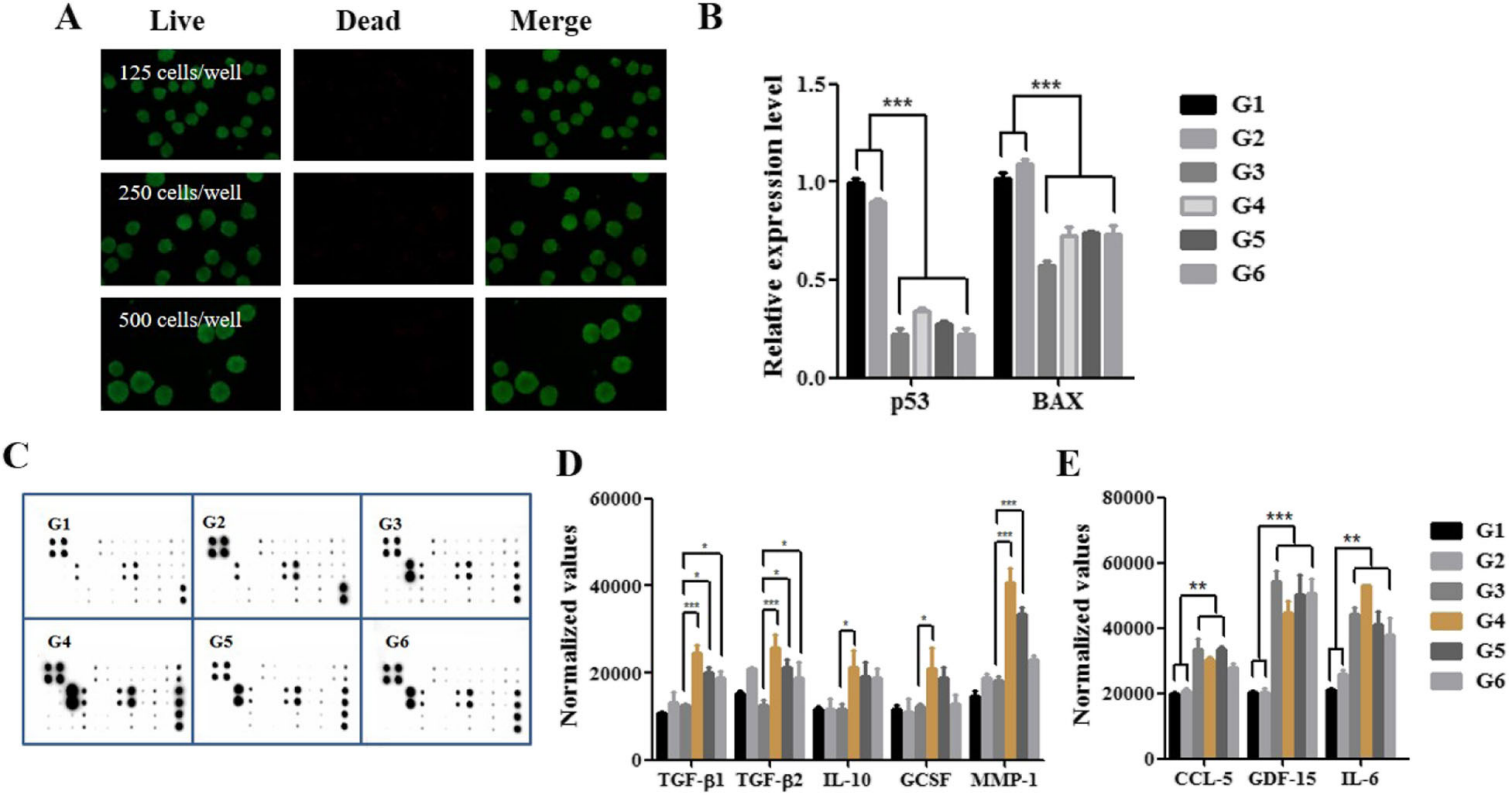
Matrilin-3 priming and culture condition optimization. **a** Live/dead assay. **b** mRNA expression of the apoptosis markers p53 and BAX. **c** Human cytokine array analysis was performed to measure the growth factors and cytokines in the conditioned medium of Ad-MSCs and primed Ad-MSCs. **d** Expression of TGF-β1, TGF-β2, IL-10, GCSF, and MMP-1 levels normalized to positive control. **e** Expression of CCL-5, GDF-15, and IL-6 levels normalized to positive control. G1, Ad-MSC monolayer; G2, matrilin-3-primed Ad-MSC monolayer; G3, Ad-MSC spheroid (125 cells/microwell); G4, matrilin-3-primed Ad-MSC spheroid (125 cells/microwell); G5, matrilin-3-primed Ad-MSC spheroid (250 cells/microwell); G6, matrilin-3-primed Ad-MSC spheroid (500 cells/microwell). Statistically significant expression at *p < 0.05, **p < 0.01, ***p < 0.001

Culture conditions were optimized with monolayers and spheroids based on apoptosis and secretome analysis. Ad-MSCs were cultured on the EZSPHERE platform at different cell seeding densities: 3 × 10^5^ cells/2400 microwells (125 cells/microwell), 6 × 10^5^ cells/2400 microwells (250 cells/microwell), and 12 × 10^5^ cells/2400 microwells (500 cells/microwell). The single cell suspension of Ad-MSCs formed uniform spheroids after 24 h of culture. The viability of the Ad-MSC spheroids was analyzed by live/dead assay. The cells on the surface of spheroids were viable, and no dead cells were observed (Fig. 2a). The mRNA expression levels of the apoptotic markers p53 and BAX were not different following priming with matrilin-3 under monolayer conditions (groups 1 and 2); however, the expression levels were significantly lower in spheroid cultures compared with monolayer cultures (groups 3–6) (Fig. 2b, ***p < 0.001).

Comparisons of the Ad-MSC secretome profile were performed after priming with matrilin-3 and spheroid preparation (Fig. 2c). Matrilin-3-primed Ad-MSC spheroids (125 cells/microwell; group 4) showed a higher level of TGF-β1, TGF-β2, interleukin-10 (IL-10), granulocyte colony-stimulating factor (G-CSF), and MMP1 compared to all the groups (Fig. 2d). Among the spheroid groups (groups 3 to 6), the secretion level was reduced with an increase in the cell number per microwell. The effects of matrilin-3 priming were minimal under monolayer conditions (groups 1 and 2) compared with spheroid conditions (groups 2 to 6). In addition, the levels of chemokine (C-C motif) ligand 5 (CCL-5), growth differentiation factor 15 (GDF-15), and IL-6 were higher in Ad-MSC spheroid cultures (groups 3 to 6) than in Ad-MSC monolayer cultures (groups 1 and 2) (Fig. 2e).

### Recovery of dNP cells in an indirect co-culture system

**Fig. 3:**
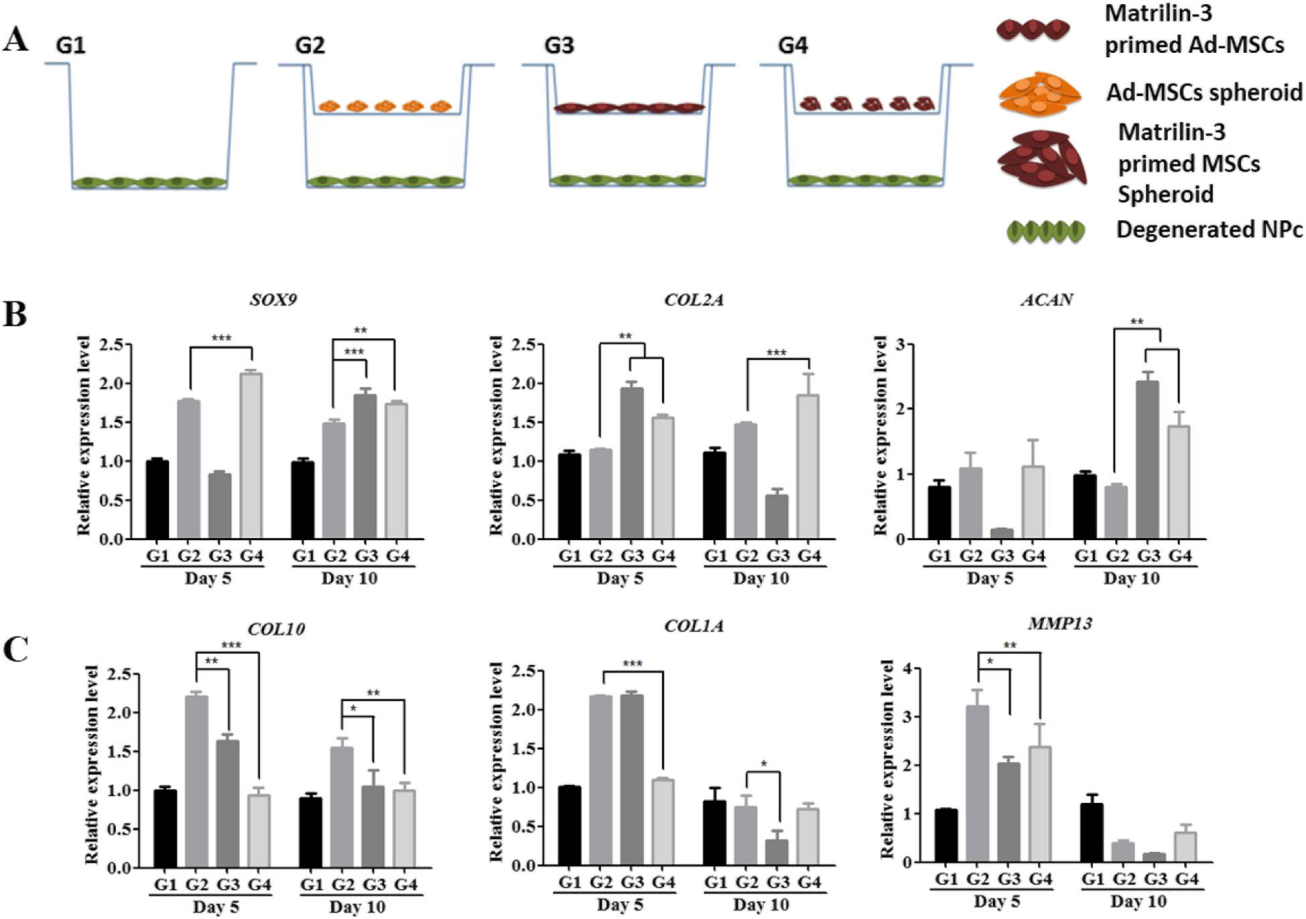
Recovery of dNP cells. **a** Schematic diagram of the indirect co-culture. **b** mRNA expression of the chondrogenic markers SOX9, collagen 2, and aggrecan in dNP cells. **c** mRNA expression of the hypertrophic markers collagen 10, collagen 1, and MMP13 in dNP cells. G1, dNP cells only; G2, Ad-MSC spheroids and dNP cells; G3, matrilin-3-primed Ad-MSC monolayer and dNP cells; G4, matrilin-3-primed Ad-MSC spheroids and dNP cells. Statistically significant expression at *p < 0.05, **p < 0.01, ***p < 0.001

**Fig. 4:**
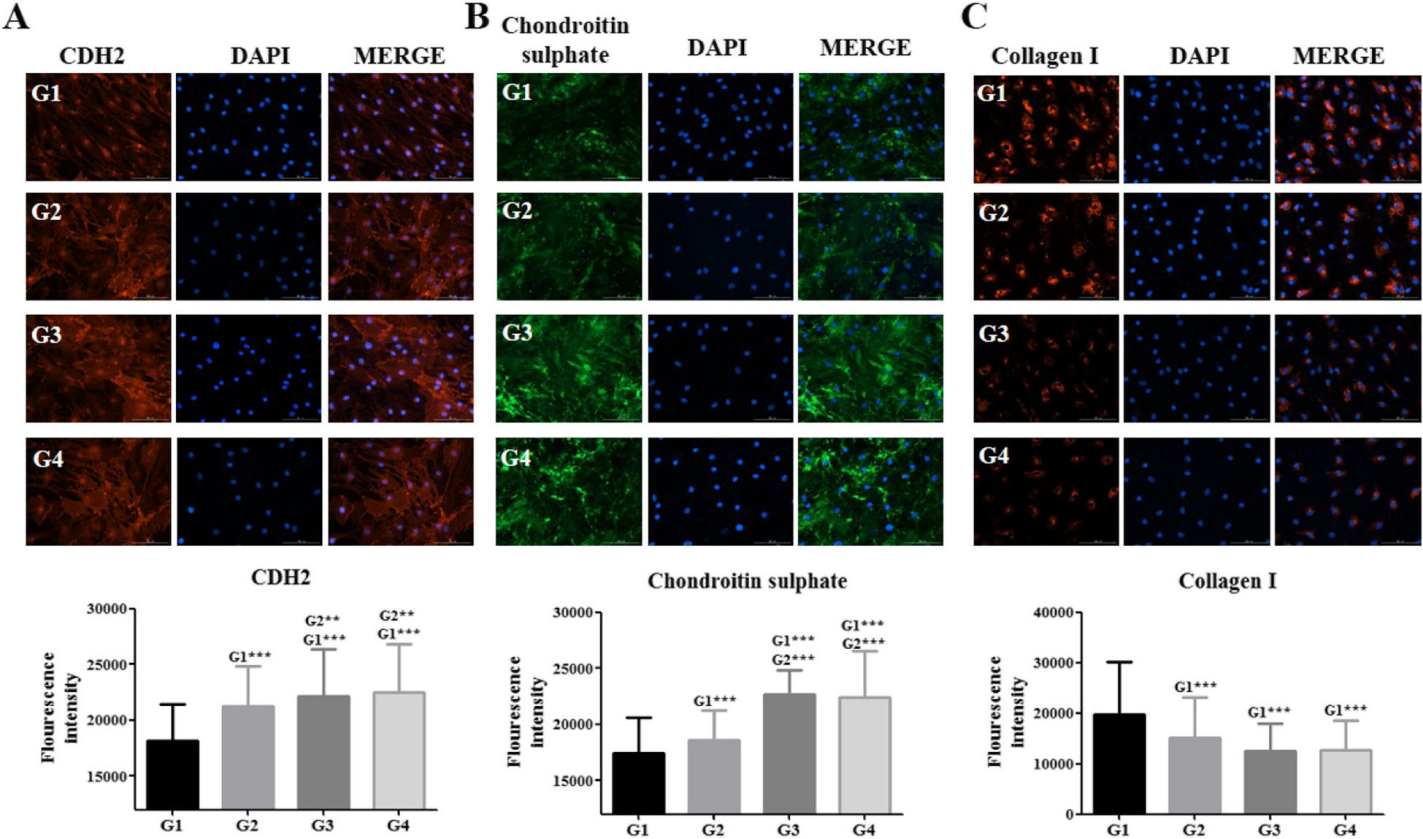
Recovery of dNP cells and ECM components. Fluorescence intensity of **a** cadherin-2, **b** chondroitin sulfate, and **c** collagen 1 in dNP cells. G1, dNP cells only; G2, Ad-MSC spheroids and dNP cells; G3, matrilin-3-primed Ad-MSC monolayer and dNP cells; G4, matrilin-3-primed Ad-MSC spheroids and dNP cells; CDH2, cadherin-2; CS, chondroitin sulfate. Statistically significant expression at *p < 0.05, **p < 0.01, ***p < 0.001

The recovery of dNP cells was performed using an indirect co-culture system (Figs. 3 and 4, Supplementary Fig. 2). The expression of SOX9 was increased in dNP cells co-cultured with Ad-MSC spheroids on day 5 and in all co-cultured groups on day 10. Collagen 2 expression was increased in dNP cells co-cultured with matrilin-3-primed Ad-MSCs on day 5 (**p < 0.01), and on day 10, the expression was increased in dNP cells cultured with Ad-MSC spheroids (***p < 0.001). The expression of aggrecan was increased on day 10 in dNP cells co-cultured with matrilin-3-primed Ad-MSCs (**p < 0.01). The stable and marked expression of SOX9, collagen 2, and aggrecan was observed in dNP cells co-cultured with matrilin-3-primed Ad-MSC spheroids compared with Ad-MSC spheroids (Fig. 3b).

The mRNA expression levels of the hypertrophic marker collagen 10, collagen 1, and MMP13 were increased in dNP cells co-cultured with Ad-MSCs (Fig. 3c). Interestingly, the expression of collagen 10 was significantly lower in dNP cells co-cultured with matrilin-3-primed Ad-MSC monolayer on day 5 (**p < 0.01) and on day 10 (*p < 0.05) and matrilin-3-primed Ad-MSC spheroids on day 5 (***p < 0.001) and on day 10 (**p < 0.01). Collagen 1 expression was significantly lower in dNP cells co-cultured with matrilin-3-primed Ad-MSC spheroids. Similarly, MMP13 expression was reduced in dNP cells co-cultured with matrilin-3-primed Ad-MSC monolayer (*p < 0.05) and matrilin-3-primed Ad-MSC spheroids (**p < 0.001) on day 5 (Fig. 3c).

Immunocytochemistry was performed, and fluorescence intensities were analyzed using ImageJ, which revealed the increased expression of cadherin-2 (CDH2) in dNP cells co-cultured with matrilin-3-primed Ad-MSC monolayer and spheroids (Fig. 4a). Furthermore, a strong fluorescence intensity for chondroitin sulfate in dNP cells co-cultured with matrilin-3-primed Ad-MSC monolayer and spheroids was observed (***p < 0.001) (Fig. 4b). Collagen 1 fluorescence intensity was reduced in dNP cells co-cultured with matrilin-3-primed Ad-MSC monolayer and spheroids; however, it was not statistically significant (Fig. 4c).

### T2 MRI and histological analyses of disc regeneration following implantation in a rabbit model

**Fig. 5:**
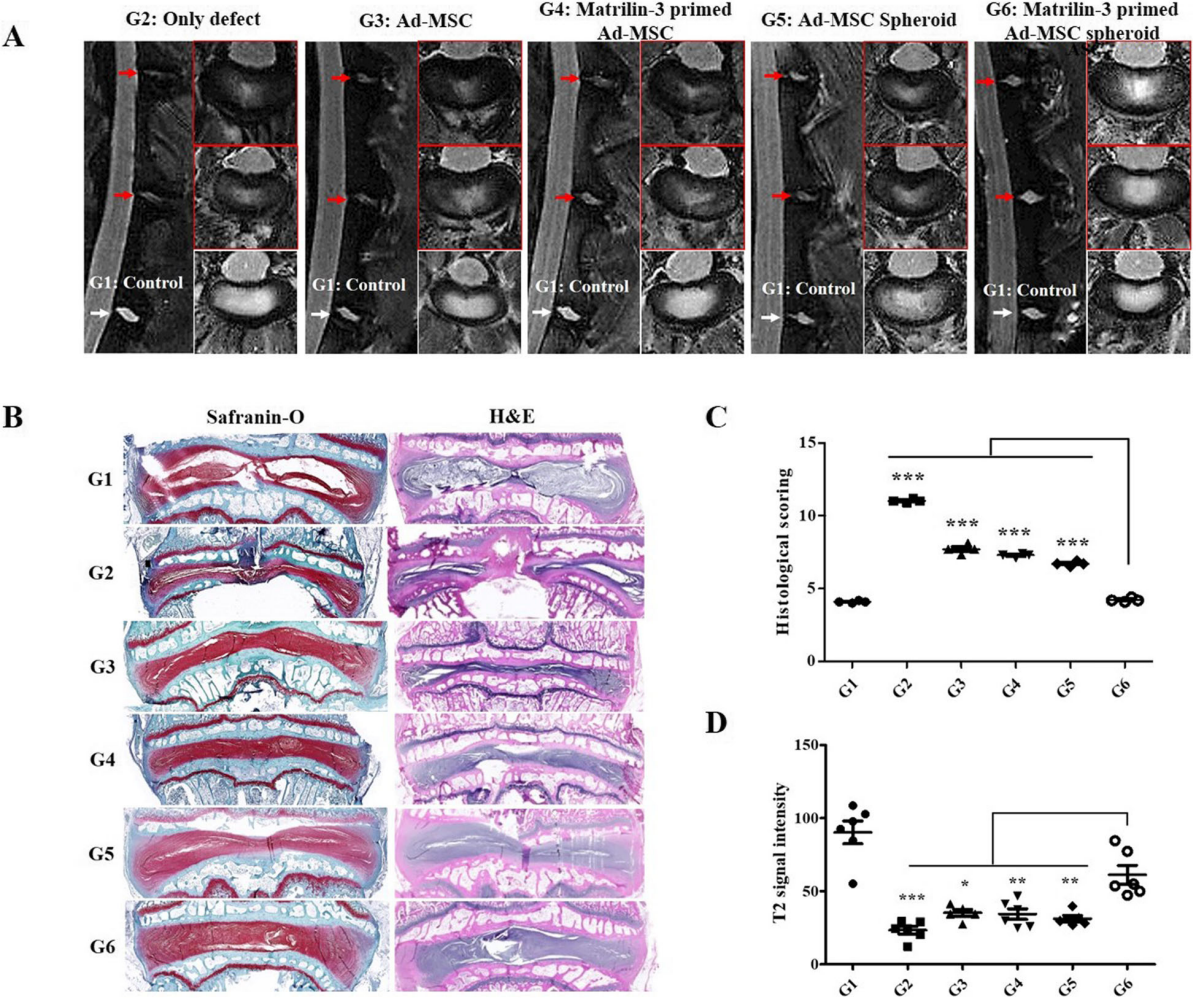
T2-weighted MRI and histological analysis for assessing disc regeneration in a rabbit model. **a** Representative T2-weighted MRI scans of the sham, single Ad-MSC (ASC), matrilin-3 (MATN3) + single ASC, ASC spheroid, and MATN3 + ASC spheroid groups. **b** Disc regeneration was assessed by Masson’s trichrome staining. The results revealed that disc regeneration was the highest in the matrilin-3-primed ASC spheroid group. **c** The histological score for disc regeneration was the highest in the matrilin-3-primed ASC spheroid group. **d** The T2 signal intensity was the highest in the matrilin-3-primed ASC spheroid group, suggesting that the matrilin-3-primed ASC spheroid was the most effective for IVD regeneration (*n* = 7). G1, control; G2, only defect; G3, single Ad-MSC; G4, matrilin-3-primed single Ad-MSC; G5, Ad-MSC spheroid; G6, matrilin-3-primed Ad-MSC spheroid. ***p < 0.001, **p < 0.01, *p < 0.05

After 12 weeks of implantation, MRI and histologic analyses were performed to evaluate the regenerative efficacy of each degenerated disc. The signal intensity of the NP was obtained from the T2-weighted MRI scans of experimental spines (sham, only defect, single Ad-MSC, matrilin-3-primed single Ad-MSC, Ad-MSC spheroid, matrilin-3-primed Ad-MSC spheroid) after sacrifice (Fig. 5a, b). The T2 signal intensity was significantly lower in the single Ad-MSC, matrilin-3-primed single Ad-MSC, and Ad-MSC spheroid groups than in the matrilin-3-primed Ad-MSC spheroid group. The T2 signal intensity of the matrilin-3-primed Ad-MSC spheroid group was increased compared with that of the matrilin-3-primed single Ad-MSC group (*p* < 0.05) and non-matrilin-3-primed spheroid group (*p* < 0.01). In comparison with the only defect group, the matrilin-3-primed Ad-MSC spheroid group had significantly higher hydration levels in the punctured discs (*p* < 0.001) (Fig. 5c).

After MRI analysis, all separated discs were evaluated by gross and histological analysis. No osteophytes were detected in all the groups, and abnormal phenomena were not observed in all experimental rabbits. Histological analysis by safranin-O staining revealed the presence of a proteoglycan matrix in the NP of the disc. Histological scores were significantly higher in the matrilin-3-primed Ad-MSC spheroid group than in the other groups (Fig. 5b). The results indicated that the combined use of matrilin-3 and 3D spheroids could provide the highest level of disc regeneration.

## Discussion

MSCs are considered to be the most attractive candidates for the restoration or regeneration of the degenerated disc [13, 19, 20, 32]. In our previous study, we have utilized of two biologically active ingredients, i.e., matrilin-3 and Ad-MSCs to regenerate defective cartilage [30]. Development of therapeutic model with single biological entity is essential to improve the Ad-MSCs research and translation to clinical use. With this contest, we have developed a therapeutic model constituting priming Ad-MSCs using matrilin-3 and spheroid formation. Here in the present study, final product contains only one biologically active ingredient, i.e., matrilin-3 primed Ad-MSCs either in single cell suspension or in spheroid form and investigated the effects of matrilin-3 priming and spheroid generation of Ad-MSCs in vitro and in vivo. The primary findings of this study were as follows: (i) the optimal conditions for the generation of matrilin-3-primed Ad-MSC spheroids were 10 ng/ml matrilin-3, 5 days of culture, and spheroid generation at a cell density of 125 cells/well; (ii) matrilin-3-primed Ad-MSC spheroids restored dNP cells in a co-culture system; and (iii) matrilin-3-primed Ad-MSC spheroids repaired the degenerated disc in a rabbit model.

We have used tissue specific extracellular matrix protein matrilin-3 as a priming agent to “stimulate or remember” Ad-MSCs transition to new microenvironment. Priming would mimic as microenvironmental stimuli at in vitro condition and avoiding the need of in vivo activation of MSCs. To generate matrilin-3-primed Ad-MSCs for in vitro and in vivo analyses, we determined the optimal conditions including optimal concentration of matrilin-3, optimal duration of matrilin-3 priming, and optimal culture conditions to improve Ad-MSC efficacy in disc repair. The priming effects of recombinant matrilin-3 on the proliferation of Ad-MSCs and its cytotoxicity were assessed. Both live/dead staining and CCK-8 assay demonstrated the cytocompatibility of matrilin-3, and it did not affect the proliferation of Ad-MSCs in the tested range (10 to 50 ng/ml) over 5 days of culture.

Although matrilin-3 priming did not affect SOX9 mRNA expression, matrilin-3 priming to Ad-MSCs enhanced the secretion of chondrogenic markers such as collagen 2 and aggrecan. Notably, matrilin-3 exhibited concentration-dependent and culture duration-dependent effects on Ad-MSCs. On culture day 5, Ad-MSCs primed with matrilin-3 at 10 ng/ml resulted in the highest collagen 2 and aggrecan mRNA expression levels. The mRNA expression of collagen 2 and aggrecan was significantly lower in Ad-MSCs primed with 20 ng/ml and 50 ng/ml matrilin-3 compared with those primed with 10 ng/ml matrilin-3. Therefore, the priming of Ad-MSCs with matrilin-3 at 10 ng/ml for 5 days was performed in further analyses. Culture conditions were optimized by comparing the monolayer and spheroid culture secretome. We prepared spheroids using matrilin-3-primed and non-primed Ad-MSCs. Live/dead assay showed that the cells on the outer surface of spheroids were viable. In addition, mRNA markers such as p53 and BAX were not significantly different. These results suggest that priming and spheroid preparation did not induce apoptosis in Ad-MSCs. Furthermore, we cultured the Ad-MSC monolayer and spheroids in serum-deprived medium for 2 days and collected the conditioned medium for cytokine array analysis. Interestingly, matrilin-3-primed Ad-MSC spheroids (125 cells/microwell) showed the significantly increased secretion of regenerative factors such as TGF-β1, TGF-β2, and MMP1, anti-inflammatory factors such as IL-10, and the endogenous stem cell mobilization factor G-CSF. The increase in the cell number/microwell (250 cells/microwell and 500 cells/microwell) during spheroid preparation reduced the secretion of the above factors. On the other hand, CCL-5, GDF-15, and IL-6 secretion levels were significantly higher in spheroid cultures compared with monolayer cultures. The results suggest that matrilin-3 could induce the secretion of these key factors from Ad-MSCs. In addition, matrilin-3 primed Ad-MSCs spheroids could promote anti-inflammatory and anabolic effects and enhance the restoration of dNP cells [31, 33–40].

We further investigated the effect of matrilin-3-primed Ad-MSCs on the recovery of dNP cells in an indirect co-culture system. Ad-MSC spheroids were able to induce only SOX9 mRNA expression in dNP cells. Furthermore, matrilin-3-primed Ad-MSCs exhibited a duration-dependent effect on collagen 2 and aggrecan mRNA expression in dNP cells. On the other hand, matrilin-3-primed Ad-MSC spheroids induced the high and stable mRNA expression levels of SOX9, collagen 2, and aggrecan in dNP cells. Moreover, both the matrilin-3-primed Ad-MSC monolayer and spheroid co-cultures significantly enhanced the anabolic ECM factor chondroitin sulfate in dNP cells. CDH2 is well known for maintaining the NP cell morphology [41]. Co-culture with both primed and non-primed Ad-MSCs increased the expression of CDH2, suggesting the recovery of cell morphology. In addition, co-culture with Ad-MSCs decreased collagen 1 expression. The overall results suggest that matrilin-3-primed Ad-MSCs could enhance the recovery of dNP cells.

A rabbit model established by needle puncture was used to evaluate the efficacy of intradiscal injection of matrilin-3-primed Ad-MSC spheroids for protection against disc degeneration. T2-weighted MRI and histological analyses demonstrated that matrilin-3-primed Ad-MSCs suppressed the acute phase of disc degeneration in the rabbit model. On T2-weighted MRI scans, IVD degeneration manifests as the loss of T2 signal in the NP [15, 38]. The T2 signal was significantly lower in the single Ad-MSC, matrilin-3-primed single Ad-MSC, and Ad-MSC spheroid groups than in the matrilin-3-primed Ad-MSC spheroid group. Histological analyses also confirmed that disc structures were well preserved in the matrilin-3-primed Ad-MSC spheroid group compared with the other groups. Therefore, the effects of matrilin-3 priming appear to be valid based on T2-weighted MRI and histological analyses. The animal study confirmed that matrilin-3-primed Ad-MSC spheroids contributed to the restoration and rehydration of the annular needle puncture-induced degenerated disc of the NP.

## Conclusion

The use of MSC therapy for the treatment of IVD degeneration has attracted increased attention. We focused on developing engineered MSCs for targeted NP regeneration. In this study, we optimized the matrilin-3 priming dose, duration, and culture conditions for the preparation of Ad-MSC spheroids as a therapeutic agent for the regeneration of the degenerated disc in an animal model. The results of cytokine array and indirect co-culture analyses indicated that the use of matrilin-3-primed Ad-MSC spheroids may be an attractive therapeutic strategy for the regeneration of the degenerated disc.

## Supplementary information

**Additional file 1: Supplementary Figure 1.** MSC surface marker expression determined by flow cytometric analysis on culture day 5 according to different concentrations of matrilin-3 (MATN3). **Supplementary Figure 2.** Cell cycle analysis. A) Flow analysis of cell cycle; B) cyclin D1 mRNA expression on culture day 6. Abbreviations: Figure A) G1: Ad-MSCs; G2: Ad-MSCs + matrilin-3 (10 ng/ml); G3: Ad-MSCs + matrilin-3 (20 ng/ml); G4: Ad-MSCs + matrilin-3 (50 ng/ml); Figure C) G1: Ad-MSCs monolayer; G2: matrilin-3 primed Ad-MSCs monolayer; G3: Ad-MSCs spheroid (125 cells/microwell); G4: matrilin-3 primed Ad-MSCs spheroid (125 cells/microwell); G5: matrilin-3 primed Ad-MSCs spheroid (250 cells/microwell); G6: matrilin-3 primed Ad-MSCs spheroid (500 cells/microwell). ***p < 0.001, **p < 0.01, * *p* < 0.05. **Supplementary Figure 3.** Ad-MSC monolayer and spheroids co-cultured with dNP cells. A) mRNA expression of the chondrogenic markers SOX9, collagen 2 (COL2A), and aggrecan (ACAN) in Ad-MSCs. B) mRNA expression of the hypertrophic markers collagen 10, collagen 1 (COL1A), and MMP13 in Ad-MSCs. Abbreviations: G2: Ad-MSC spheroids and dNP cells; G3: matrilin-3-primed Ad-MSC monolayer and NP cells; G4: matrilin-3-primed Ad-MSC spheroids and dNP cells. ***p < 0.001, **p < 0.01, *p < 0.05. **Supplementary Figure 4.** Viability and distribution of Ad-MSC spheroids after mixing with hyaluronic acid.

## Data Availability

All materials are available by the corresponding author.

## Abbreviations

TGF-β: Transforming growth factor beta
BMP: Bone morphogenetic protein p53 and BAX
MMP: Matrix metallopeptidase
CCL-5: Chemokine (C-C motif) ligand 5
GDF-15: Growth/differentiation factor 15
IL-6: Interleukin 6
