# Supplementary material for: Efficacy of matrilin-3-primed adipose-derived mesenchymal stem cell spheroids in a rabbit model of disc degeneration
Source: Stem Cell Res Ther. 2020 Aug 24;11:363. doi: 10.1186/s13287-020-01862-w (PMC7444036; doi:10.1186/s13287-020-01862-w)
Supplement: Supplementary file 1 — Additional file 1: Supplementary Figure 1. MSC surface marker expression determined by flow cytometric analysis on culture day 5 according to different concentrations of matrilin-3 (MATN3). Supplementary Figure 2. Cell cycle analysis. A) Flow analysis of cell cycle; B) cyclin D1 mRNA expression on culture day 6. Abbreviations: Figure A) G1: Ad-MSCs; G2: Ad-MSCs + matrilin-3 (10 ng/ml); G3: Ad-MSCs + matrilin-3 (20 ng/ml); G4: Ad-MSCs + matrilin-3 (50 ng/ml); Figure C) G1: Ad-MSCs monolayer; G2: matrilin-3 primed Ad-MSCs monolayer; G3: Ad-MSCs spheroid (125 cells/microwell); G4: matrilin-3 primed Ad-MSCs spheroid (125 cells/microwell); G5: matrilin-3 primed Ad-MSCs spheroid (250 cells/microwell); G6: matrilin-3 primed Ad-MSCs spheroid (500 cells/microwell). ***p < 0.001, **p < 0.01, * p < 0.05. Supplementary Figure 3. Ad-MSC monolayer and spheroids co-cultured with dNP cells. A) mRNA expression of the chondrogenic markers SOX9, collagen 2 (COL2A), and aggrecan (ACAN) in Ad-MSCs. B) mRNA expression of the hypertrophic markers collagen 10, collagen 1 (COL1A), and MMP13 in Ad-MSCs. Abbreviations: G2: Ad-MSC spheroids and dNP cells; G3: matrilin-3-primed Ad-MSC monolayer and NP cells; G4: matrilin-3-primed Ad-MSC spheroids and dNP cells. ***p < 0.001, **p < 0.01, *p < 0.05. Supplementary Figure 4. Viability and distribution of Ad-MSC spheroids after mixing with hyaluronic acid. [file 13287_2020_1862_MOESM1_ESM.docx]

**
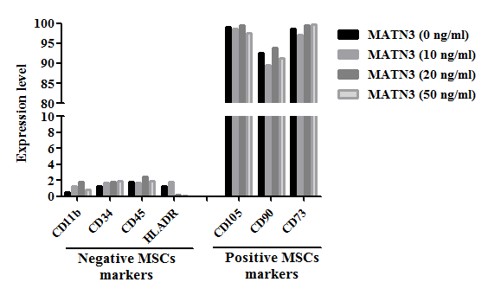
**

**Supplementary Figure 1.** MSC surface marker expression determined by flow cytometric analysis on culture day 5 according to different concentrations of matrilin-3 (MATN3).

**
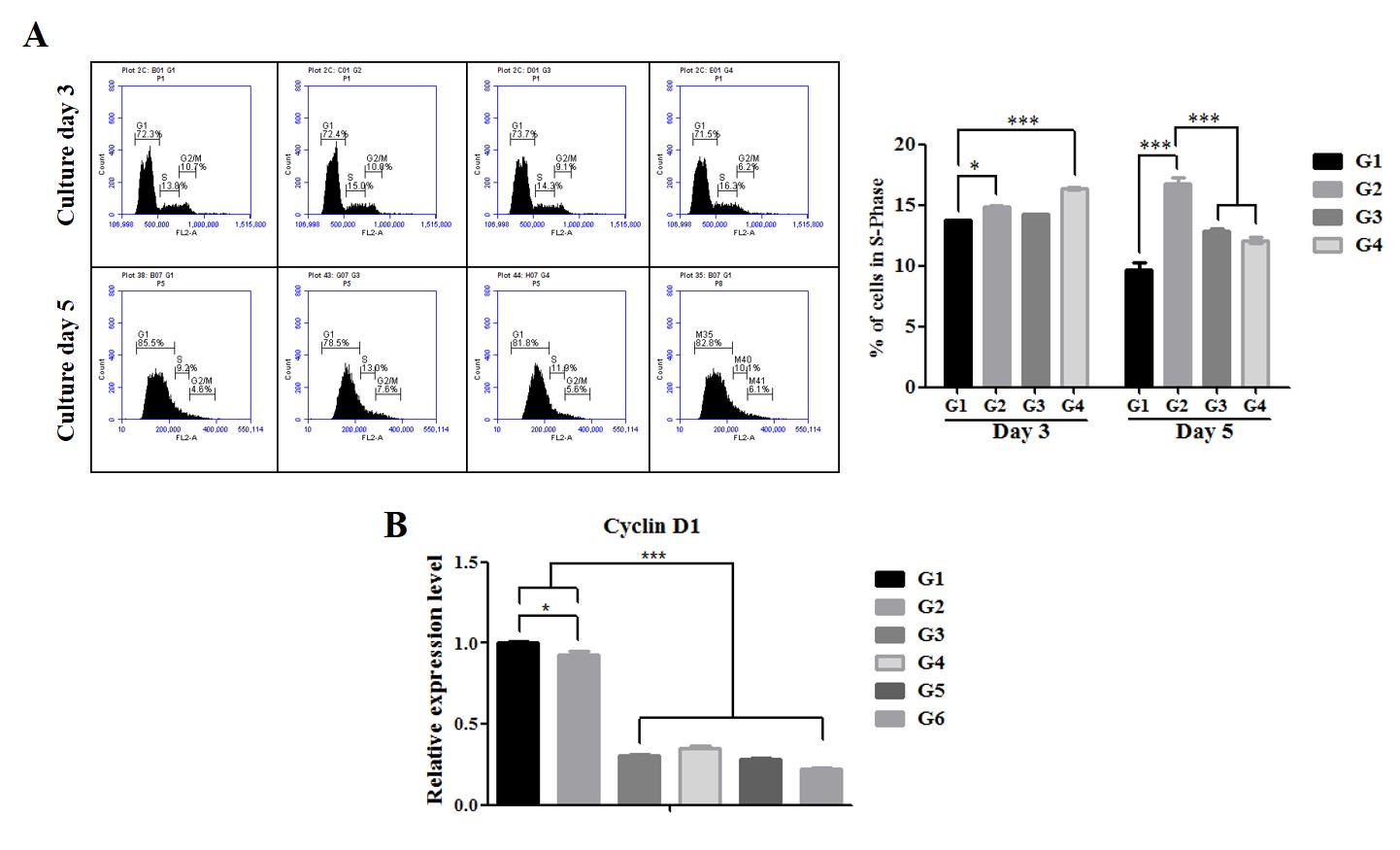
**

**Supplementary Figure 2.** Cell cycle analysis. A) Flow analysis of cell cycle; B) cyclin D1 mRNA expression on culture day 6. Abbreviations: Figure A) G1: Ad-MSCs; G2: Ad-MSCs + matrilin-3 (10 ng/ml); G3: Ad-MSCs + matrilin-3 (20 ng/ml); G4: Ad-MSCs + matrilin-3 (50 ng/ml); Figure C) G1: Ad-MSCs monolayer; G2: matrilin-3 primed Ad-MSCs monolayer; G3: Ad-MSCs spheroid (125 cells/microwell); G4: matrilin-3 primed Ad-MSCs spheroid (125 cells/microwell) ; G5: matrilin-3 primed Ad-MSCs spheroid (250 cells/microwell); G6: matrilin-3 primed Ad-MSCs spheroid (500 cells/microwell). ***p<0.001, **p<0.01, * p<0.05.

**
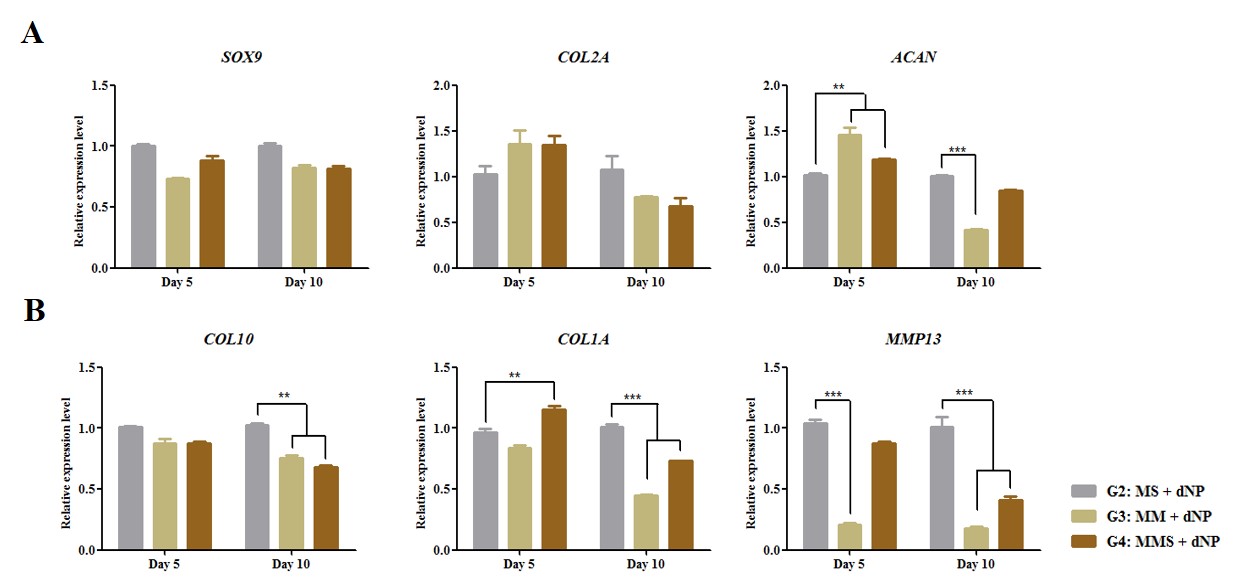
Supplementary Figure 3.** Ad-MSC monolayer and spheroids co-cultured with dNP cells. A) mRNA expression of the chondrogenic markers SOX9, collagen 2 (COL2A), and aggrecan (ACAN) in Ad-MSCs. B) mRNA expression of the hypertrophic markers collagen 10, collagen 1 (COL1A), and MMP13 in Ad-MSCs. Abbreviations: G2: Ad-MSC spheroids and dNP cells; G3: matrilin-3-primed Ad-MSC monolayer and NP cells; G4: matrilin-3-primed Ad-MSC spheroids and dNP cells. ***p < 0.001, **p < 0.01, *p < 0.05.

**
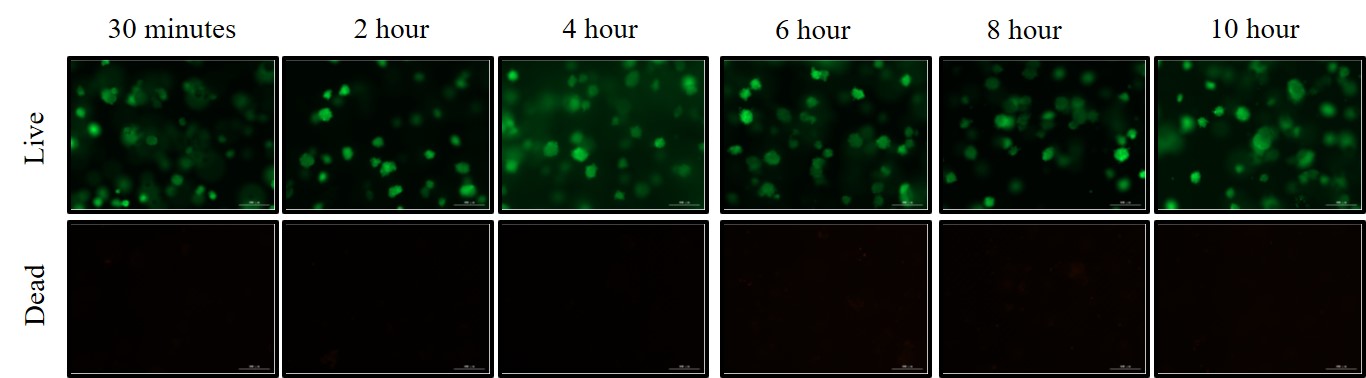
**

**Supplementary Figure 4.** Viability and distribution of Ad-MSC spheroids after mixing with hyaluronic acid.
